# Editorial: Social influences on ontogenetic development

**DOI:** 10.3389/fpsyg.2026.1805815

**Published:** 2026-03-23

**Authors:** Julia M. Rodriguez Buritica, Wouter van den Bos, Ben Eppinger, Simon K. Ciranka

**Affiliations:** 1 Department of Psychology, University of Greifswald, Greifswald, Germany; 2 Department of Psychology, Berlin School of Mind and Brain, Humboldt-Universität zu Berlin, Berlin, Germany; 3 Science of Intelligence, Research Cluster of Excellence, Berlin, Germany; 4 Department of Psychology, University of Amsterdam, Amsterdam, Netherlands; 5 Amsterdam Brain and Cognition, University of Amsterdam, Amsterdam, Netherlands; 6 Center for Adaptive Rationality, Max-Planck-Institut für Bildungsforschung, Berlin, Germany; 7 Department of Psychology, Technische Universität, Dresden, Germany; 8 Department of Psychology, Concordia University, Montreal, QC, Canada

**Keywords:** adolescence, child development, intervention, lifespan, online violence, play, risk taking, social connectedness

The nature of social influences changes markedly across development. During childhood, family-based social interactions dominate, whereas during adolescence, peer relationships become more important. During adulthood, social networks often shrink, and interactions with romantic partners become more prominent. Social influences, offline and online, provide pillars of adaptive functioning throughout the lifespan: they shape learning, emotion regulation, social attitudes, behavior, and communication. However, social influences may also be maladaptive. For example, persistent negative social experiences during adolescence may foster antisocial behavior and increase vulnerability to psychological disorders. Social influences on the ontogenetic development of adaptive functioning remain inadequately explored; this Research Topic sought to address this gap. The articles in this topic fall into three themes: social influence among children and adolescents, assessment and development of social skills in children, and connectedness across the lifespan. Together, they demonstrate the promises and pitfalls of social influence on adaptive behavior across the lifespan.

## Social influence in children and adolescents

Prior to school entry, children's social competencies are primarily shaped within the family context (Anthony et al., 2005) with parental—particularly maternal—engagement playing a central role (Gryczkowski et al., 2010). Wu et al. examined profiles of paternal and maternal involvement in physical play, didactic activities, socialization, and caregiving among 3-6-year-olds. Across all activity domains, mothers exhibited higher levels of involvement than fathers. Importantly, high-involvement for both parents was associated with superior social skills and cognitive control compared with less involvement, but not related to differences in problem behavior. These findings suggest that early social influences are distributed across caregivers and that high involvement supports children's social and cognitive development in different caregiving systems.

During the school years, social influences on behavior become increasingly complex. With the onset of adolescence, peers gain importance alongside the family context (Blakemore and Mills, 2014; Steinberg and Monahan, 2007), and negative social experiences such as exclusion exert lasting effects on social behavior (Leary et al., 2003). Lorenz et al. investigated the interaction between social exclusion, social acceptance, and antisocial risk-taking in adolescents aged 12–16 years. Social exclusion was not uniformly associated with antisocial risk-taking, but moderated by in-classroom acceptance. Well-integrated adolescents responded to exclusion by reducing antisocial risk-taking, whereas poorly accepted adolescents increased risk-taking that could harm others. These findings indicate that negative adolescent social experiences may lead to antisocial responses to exclusion, underscoring the role of classroom social integration.

The study by Molenaar et al. complements this perspective by including the online world. Traditionally, research has focused on peers and classroom contexts, rightly so, but adolescent development also happens in the online world. In a sample of youth aged 16 to 24, the exposure to violent social media content was associated with real-life violence. Furthermore, this association was partially moderated by developmental stage, suggesting that it is stronger among younger adolescents. Although these findings are cross-sectional and do not establish causality, they highlight a strong association between adolescents' online and offline worlds and underscore the need to understand the social dynamics between online content and offline behaviors.

## Assessing and fostering social skills in children

According to Vygotskii (1978), “play creates a zone of proximal development of the child” (p. 102), in which children practice activities they cannot perform in real life. Play helps to practice social interactions and serves as a scaffold for sociocognitive development (Alberts and Pyclik, 2025). Veraksa et al., provide an evaluation of the “play matrix tool”. In it, Veraksa et al. show that indicators of play in children aged 5–6 are correlated with other validated indices of executive functioning. This work demonstrates the rich interplay between socio-emotional and cognitive development and provides an ecologically valid instrument for studying both in children.

Despite the potential of early socioemotional skills as discussed by Veraksa et al. to shape a child's future positively, educational institutions often do not foster them, but this is desperately needed, argue (Lechner et al.) Their developed puppet-theater intervention, the Papillo 6–9 program, taught children aged 6–9, among other topics, about social emotions like envy, shame, guilt, and pride. In this longitudinal study, they demonstrate its overall effectiveness. Lechner et al. state that fostering social skills can build children's resilience in stressful situations and demonstrate that this works; so, what are policymakers waiting for?

## Social connectedness across the lifespan

The child-mother relationship is among the most enduring relationships of childhood affecting psychological wellbeing for both parties (Stafford et al., 2016); however, it remains poorly understood. Schoenert et al. investigate reciprocal social support between mothers (40–87 years) and their adult children (20–49 years). By asking them about their attitudes toward one another, Schoenert et al. reveal that a sense of obligation and reciprocity affects mutual support. However, reciprocity and relationship quality mattered more for mothers, while obligation had a stronger effect for children. This demonstrates a reciprocal asymmetry between mothers and children that may challenge evolutionary arguments that explain human relationships through reciprocity (Schmid et al., 2021).

Rauers et al. study how relationships between adult partners shape communication later in life. The authors asked younger and older adults to explain concepts to their partners with as few words as possible. Efficiency was investigated using references to shared experiences between partners as a strategy to alleviate cognitive resource limitations during communication. When target concepts were difficult to explain, both age groups used such idiosyncratic cues to increase communication efficiency. This suggests that shared knowledge between romantic partners facilitates collaborative communication across the adult lifespan. Future research could focus on the communication between children and their parents and how it affects subsequent social development.

## Summary and conclusion

Taken together, this issue highlights the rich social experiences during lifespan development. Social influences operate through interindividual-interactions, family involvement, social integration within school and among peers, and the online world. They shape developmental trajectories for better or worse, possibly depending on the socioemotional skills that children develop early on. These can, and should, be fostered across the lifespan.
